# Antagonism of Sorafenib and Regorafenib actions by platelet factors in hepatocellular carcinoma cell lines

**DOI:** 10.1186/1471-2407-14-351

**Published:** 2014-05-21

**Authors:** Rosalba D’Alessandro, Maria G Refolo, Catia Lippolis, Grazia Giannuzzi, Nicola Carella, Caterina Messa, Aldo Cavallini, Brian I Carr

**Affiliations:** 1 Laboratory of Biochemistry, National Institute for Digestive Diseases, IRCCS “Saverio de Bellis”, Via Turi 27, 70013, Castellana Grotte, BA, Italy; 2 Transfusion Medicine Center, “S. Maria degli Angeli” Hospital, via Cappuccini 7, 70017 Putignano, BA, Italy

**Keywords:** Regorefenib, Platelets, HCC, Apoptosis, Growth, Invasion

## Abstract

**Background:**

Platelets are frequently altered in hepatocellular carcinoma (HCC) patients. Platelet lysates (hPL) can enhance HCC cell growth and decrease apoptosis. The aims were to evaluate whether hPL can modulate the actions of Sorafenib or Regorafenib, two clinical HCC multikinase antagonists.

**Methods:**

Several human HCC cell lines were grown in the presence and absence of Sorafenib or Regorafenib, with or without hPL. Growth was measured by MTT assay, apoptosis was assessed by Annexin V and by western blot, and autophagy and MAPK growth signaling were also measured by western blot, and migration and invasion were measured by standard in vitro assays.

**Results:**

Both Sorafenib and Regorafenib-mediated inhibition of cell growth, migration and invasion were all antagonized by hPL. Drug-mediated apoptosis and decrease in phospho-ERK levels were both blocked by hPL, which also increased anti-apoptotic phospho-STAT, Bax and Bcl-xL levels. Preliminary data, obtained with epidermal growth factor (EGF) and insulin-like growth factor-I (IGF-I), included in hPL, revealed that these factors were able to antagonized Sorafenib in a proliferation assay, in particular when used in combination.

**Conclusions:**

Platelet factors can antagonize Sorafenib or Regorafenib-mediated growth inhibition and apoptosis in HCC cells. The modulation of platelet activity or numbers has the potential to enhance multikinase drug actions.

## Background

Platelet activity has been known for a long time to be altered in the presence of cancer, with venous thrombosis being recognized in association with occult malignancy [1,2]. In addition to the effects of cancer on platelet actions in blood clotting, platelets have been recognized to be involved in cancer development, progression and metastasis [3-8]. Platelet levels have been shown to impact prognosis in several cancers, including those of the ovary, kidney, colon, lung and pancreas [9-14]. Furthermore, whereas hepatocellular carcinoma (HCC) most typically arises on the basis of cirrhosis, with its frequently associated splenomegaly and thrombocytopenia, normal or elevated platelet levels are frequently seen in large size HCCs [15-17]. We recently found that platelet extracts can stimulate HCC cell line growth in vitro, which was associated with a decrease in apoptosis [18]. We now extend those observations, by examining the effects of platelet extracts on the effects of apoptosis-inducing HCC treatment agents and report that platelet extracts can antagonize growth inhibition mediated by Sorafenib or Regorafenib.

## Methods

### Cells and materials

PLC/PRF/5, Hep3B and HepG2 cells were obtained from the ATCC and were cultured as previously described [19].

Recombinant human EGF was purchased from PeproTech (Rocky Hill, NJ, USA), mouse recombinant IGF-I from Calbiochem (San Diego, CA, USA) and serotonin from Sigma-Aldrich (Saint Louis, MO, USA), all the growth factors were dissolved in water.

### Platelet lysates

The platelet samples were collected from healthy volunteers. The study protocol was approved by the institutional review boards of the University of Bari and “Saverio de Bellis” Institute of Castellana G. (BA), Italy. Additionally, written informed consent was obtained from participants for the use of their blood in this study.

The platelet-rich plasma was obtained using an automated hemapheresis procedure in a local blood transfusion center. The platelets obtained from different volunteers were mixed and then divided into aliquots. Each aliquot was subjected to several freeze-thaw cycles to disrupt their membranes and release the growth factors stored in the granules (human Platelet Lysate, hPL).

### Growth assay

Proliferation assay was performed as recently described [19,20]. The cells were cultured in 1% FBS medium containing different hPL concentrations (2.5 - 3.75 × 10^7^) or equivalent percentage of FBS in presence of 1 μM (HepG2 cell line) or 2.5 μM (Hep3B and PLC/RFP/5) of Sorafenib or Regorafenib. In the same growth condition HCC cell lines were cultured in presence of EGF 10, 25 mg/ml, IGF-I 50, 100 mg/ml and serotonin 1, 10 μM with or without Sorafenib 1 μM.

### AFP measurement

Medium AFP levels were measured using an automated system (UniCel Integrated Workstations DxC 660i, Beckman Coulter, Fullerton, CA, USA) by a chemoluminescent immunometric method. Sample measurements over the calibration range were automatically re-analyzed according to manufacture’s instructions.

### Migration assay

A scratch assay was performed as previously described [19,20]. Briefly, a wound was generated with a pipette tip, after rinsing, medium containing different concentrations of hPL or equivalent percentage of FBS and 2.5 μM Sorafenib or Regorafenib was added. Photographs were taken of each well immediately and after 24 h and 48 h. The values were expressed as percentage of migration, with 100% being when the wound was completely closed. The results were representative of three independent experiments.

### Invasion assay

Cell invasion assays were performed using Matrigel (BD Transduction, San Jose, CA, USA)-coated Transwells (8 μm pore PET membrane, Millipore, Billerica, MA, USA) as previously described (19). Briefly, 2.5 μM Sorafenib or Regorafenib treated cells were suspended in low serum medium. Medium containing different hPL or FBS concentrations was added to the bottom wells. After incubation of 24 h, the invading cells were fixed and stained. The images were acquired and analyzed counting the cells with Image J Software (National Institute of Health, USA). Values obtained were expressed as fold increase of invading cells, setting the cell counts of control cells as one. Results were representative of three independent experiments.

### Apoptosis assays

#### Annexin V

The Muse Annexin V/Dead Cell Assay Kit (Millipore, Darmstadt, Germany) for quantitative analysis of live, early/late apoptotic and dead cells was used with a Muse Cell Analyzer (Millipore). Briefly, the assay utilizes Annexin V to detect PS on the external membrane of apoptotic cells. A dead cell marker (7-AAD) is also used. PLC/PRF/5 cell line, including positive and negative controls, were cultured in 1% FBS medium supplemented with a volume of hPL corresponding to 3.75 × 10^7^ platelets/ml or with an equivalent percentage of serum (control cells) for 48 h. The cells were then processed as described in the user’s guide.

#### Caspase-3/7 quantitative measurements

The Muse Caspase-3/7 kit (Millipore) permits simultaneous evaluation of apoptotic status based on Caspase-3 and -7 activation and cellular plasma membrane permeabilization (cell death). The assay provides relative percentage of cells that are live, early/late apoptotic or dead. Cells were cultured as described above and processed according to the user’s guide.

### Western blots

We analyzed the MAPK signaling and anti-apoptosis markers in Hep3B cells treated with 2.5 μM Sorafenib or Regorafenib and hPL by Western blot, as previously described [19,20]. In brief, cells were washed twice with cold PBS and then lysed in RIPA buffer (Sigma-Aldrich, Milan; Italy). After quantization of protein concentration, equal amount of protein (50 μg) were resolved on SDS–PAGE and transferred to polyvinyldifluoride (PVDF) filters. The blots were blocked with 5% (w/v) nonfat dry milk for 2 h at room temperature and then probed with primary antibody overnight at 4°C.

The primary antibodies were directed against the following proteins: ERK and phospho-ERK (P-ERK), JNK and phospho-JNK (P-JNK), p38 and phospho-p38 (P-p38), STAT3 and phospho-STAT3 (Tyr^705^, Ser^727^) (P-STAT3), AKT and phospho-AKT (P-AKT), survivin, Bcl-xL, Bax, Bim and β-actin (Cell Signaling, Beverly, MA, USA). After three washes, incubation was followed by the reaction with horseradish peroxidase-conjugated secondary antibody for 1 h at room temperature. The immunoreactive bands were visualized and analyzed using the enhanced chemiluminescence detection reagents (Cell Signaling), according to the manufacturer’s instructions, and chemiluminescence detection system (ChemiDoc XRS apparatus and software, Bio-Rad).

### Statistical analysis

GraphPad Prism 5.0 software (La Jolla, CA, USA) was used for all statistical analysis. Mann–Whitney nonparametric test was employed to assess the statistical significance of differences between two groups. For multiple comparisons was used one-way Anova test followed by appropriate post-test. P-values of <0.05 were considered statistically significant. All experiments were done in triplicate and data are presented as mean ± standard deviation (SD).

## Results

### Platelet factors antagonize drug-mediated inhibition of HCC cell growth

hPL were previously examined for the ability to stimulate human HCC cell line growth [18]. Hep3B, PLC/PRF/5 and HepG2 human HCC cell lines were treated in log phase growth with 1 μM (HepG2) - 2.5 μM (Hep3B and PLC/PRF/5) Regorafenib or Sorafenib, concentrations which are known to decrease in HCC cell proliferation [19].

**Figure 1:**
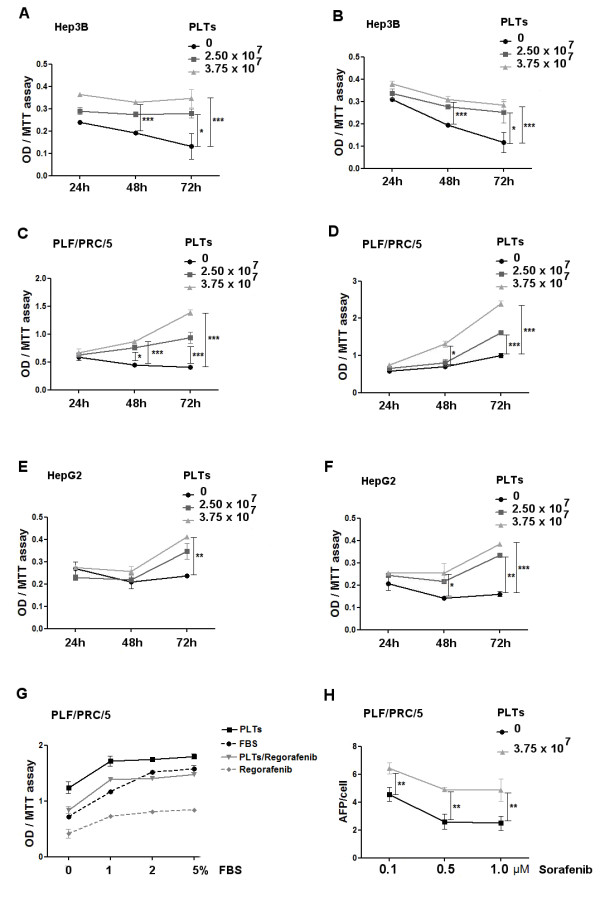
**Platelets antagonism of Sorafenib or Regorafenib mediated cell growth inhibition.** Hep3B **(A, B)**, PLC/PRF/5 **(C, D)** and HepG2 **(E, F)** cell lines were cultured in 1% FBS medium in presence of different platelet concentrations or FBS and incubated with 1–2.5 μM Sorafenib **(A, C, E)** or Regorafenib **(B, D, F)**. MTT assay was assessed after 24-72 h. (G) MTT assay performed on PLC/RFP/5 cells treated or untreated with 2.5 μM Regorafenib and cultured in different FBS concentrations (0-5%) for 48 h in presence or absence of hPL derived from 3.75 × 10^7^ platelets. **(H)** AFP levels in the cell culture medium of PLC/PRF/5 cell lines containing hPL or FBS after treatment with different Sorafenib concentrations. The results are expressed as mean ± SD. *p < 0.05; **p < 0.001; ***p < 0.0001.

Cells were also treated in the absence or presence of increasing concentrations of hPL. A significant increase of cell growth was detected in presence of hPL from 3.75 × 10^7^ platelets in all the HCC cell lines, compared with treatments with Regorafenib or Sorafenib in presence of FBS. Figure 1A-F shows the time course of these effects on the three cell lines. In order to exclude a possible FBS effects on the observed antagonism of cell growth inhibition due to drug action, PLC/RFP/5 cells treated or untreated with 2.5 μM Regorafenib were cultured in different FBS concentrations (0-5%) for 48 h in presence or absence of hPL derived from 3.75 × 10^7^ platelets.

Comparing the growth in these different conditions by MTT assay, it was clear that increasing the serum concentration more than 1% had not significant influence on PLTs antagonism (Figure 1G). Identical results were obtained with Sorafenib treatments (data not shown).

The concentrations of medium alpha-fetoprotein (AFP), an HCC cell growth marker, were also measured. We found that Sorafenib-mediated inhibition of AFP levels was also antagonized by the presence of hPL (Figure 1H).

### Effects of platelet factors on cell signaling

**Figure 2:**
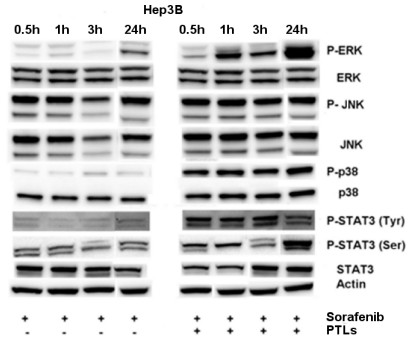
**Platelet extracts counteract the inhibitory effects of Sorafenib.** A representative Western blot of 2.5 μM Sorafenib action that inhibits RAF/MEK/ERK signaling in Hep3B cells (to the left of the figure). The same action had been observed by Regorafenib (data not shown). The presence of hPL from 3.75 × 10^7^ platelets reduced drug effects (to the right) with an increase of P-ERK, P-p38, P-STAT3, consistent with the induction of cell proliferation. No changes were observed in the phosphorylation levels of JNK.

Both Sorafenib and Regorafenib have previously been shown to cause a decrease in P-ERK levels, consequent on Raf inhibition. Here, we examined the effects of 2.5 μM Sorafenib or Regorafenib on P-ERK levels in Hep 3B cells in the absence or presence of hPL from 3.75 × 10^7^ platelets. We found that hPL caused an increase in P-ERK levels, as well as for P-p38 and P-STAT3 (Ser and Tyr). By contrast, P-JNK levels were not modified by the presence or absence of hPL (Figure 2).

### Platelet factor antagonism of drug-mediated inhibition of migration and invasion

Both Sorafenib and Regorafenib can inhibit both HCC cell migration and invasion through Matrigel membranes. Furthermore, hPL has been shown to stimulate cell motility [21]. We therefore added hPL to 2.5 μM concentrations of Sorafenib or Regorafenib that could inhibit both migration and invasion in Hep3B cells.

**Figure 3:**
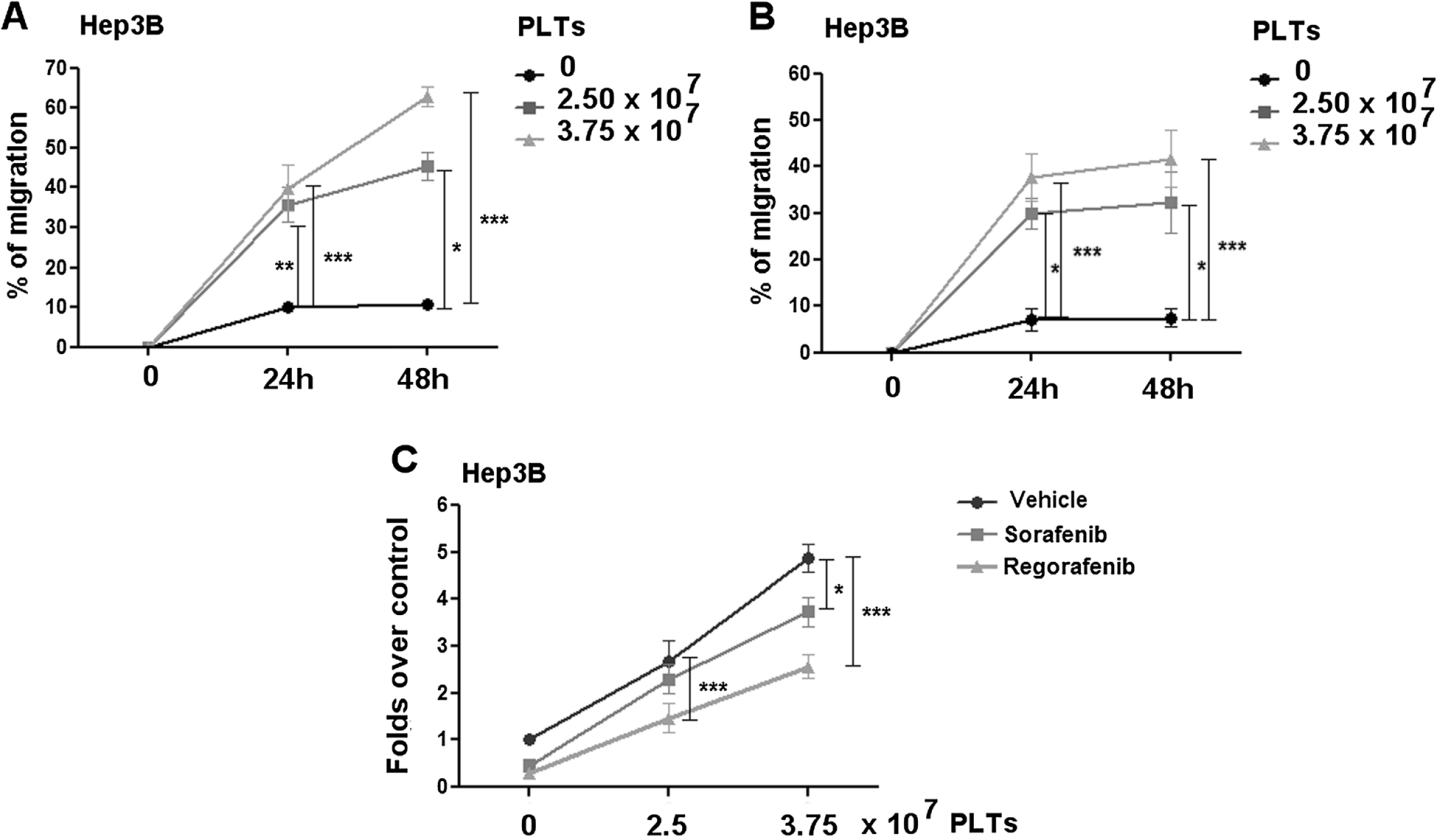
**Platelet antagonism of drug-mediated inhibition of migration and invasion.** Hep3B cell line was cultured in 1% FBS medium in presence of different platelets concentrations or FBS and incubated with 2.5-5 μM Sorafenib **(A, C)** or Regorafenib **(B, C)**. Results of cell migration **(A, B)** and invasion **(C)** were expressed as percentage of migration (100% representing the completely closed wound) and fold increase over control invading cell, respectively.

We found that hPL antagonized the inhibition by Sorafenib or Regorafenib on both migration and invasion (Figure 3A-C). Identical results were found for the other cell lines (data not shown).

### Platelet factor antagonism of drug-mediated induction of apoptosis

To evaluate the possible platelet factor mechanisms, we examined their effects on Sorafenib or Regorafenib–mediated apoptosis, since that is one major aspect of their growth-inhibitory actions.

**Figure 4:**
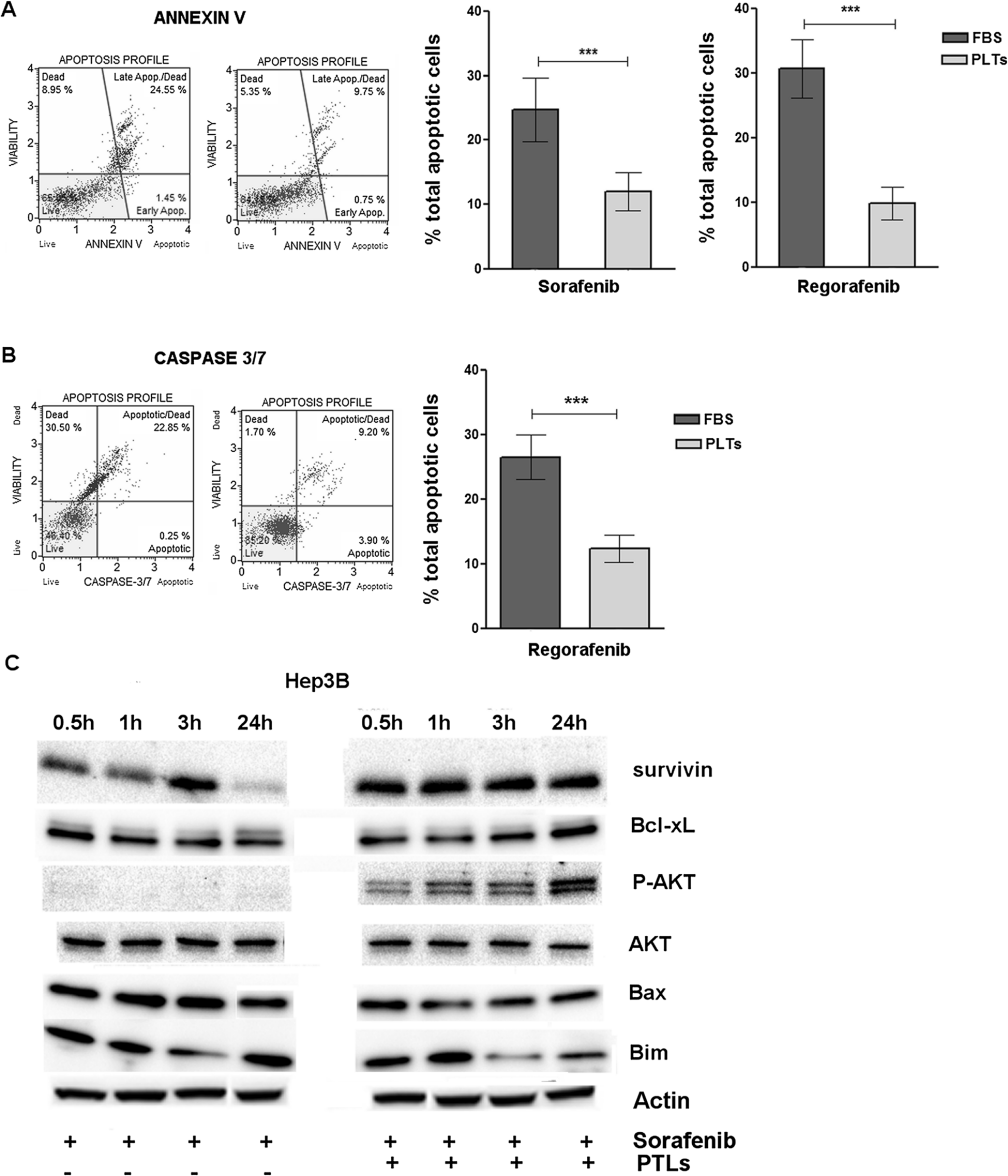
**Platelet extract antagonism of drug-mediated inhibition of apoptosis.** Apoptosis assays. On the left are shown examples of the results obtained using the Muse Annexin V kit **(A)** or Caspase-3/7 kit **(B)** to evaluate the percentage of apoptotic PLC/PRF/5 Hep3B cells treated with 2.5 μM of Regorafenib **(A-B)** or Sorafenib **(A)** and cultured whit 3.75 × 10^7^ hPL or equivalent FBS are expressed in the relative graph as the mean of three independent experiments. On the right the mean of three independent experiments is plotted in the relative graph. The results are expressed as mean ± SD. ***p < 0.0001. The anti-apoptotic effect of hPL was also evaluated at the molecular level **(C)**. The representative Western blot shows Hep3B cells treated with 2.5 μM Sorafenib with and without hPL from 3.75 × 10^7^ platelets. hPL caused increased levels of anti-apoptotic factors (survivin, Bcl-xL, P-AKT) and decreased levels of pro-apoptotic (Bax, Bim) factors.

The drug induced both an increase in Annexin V and activation of Caspase 3/7, two separated apoptosis markers. When hPL were also added to the cell medium together with drug, a pronounced and significant inhibition in apoptosis induction was found (Figure 4A-B). These results were confirmed at the protein level with an increase of survivin, Bcl-xL and P-AKT levels (anti-apoptotic factors) and a decrease of Bax and Bim levels (pro-apoptotic factors) in Hep3B cells treated with 2.5 μM Sorafenib or Regorafenib in presence of hPL from 3.75 × 10^7^ platelets (Figure 4C).

### EGF and IGF antagonize drug-mediated inhibition of HCC cell growth

**Figure 5:**
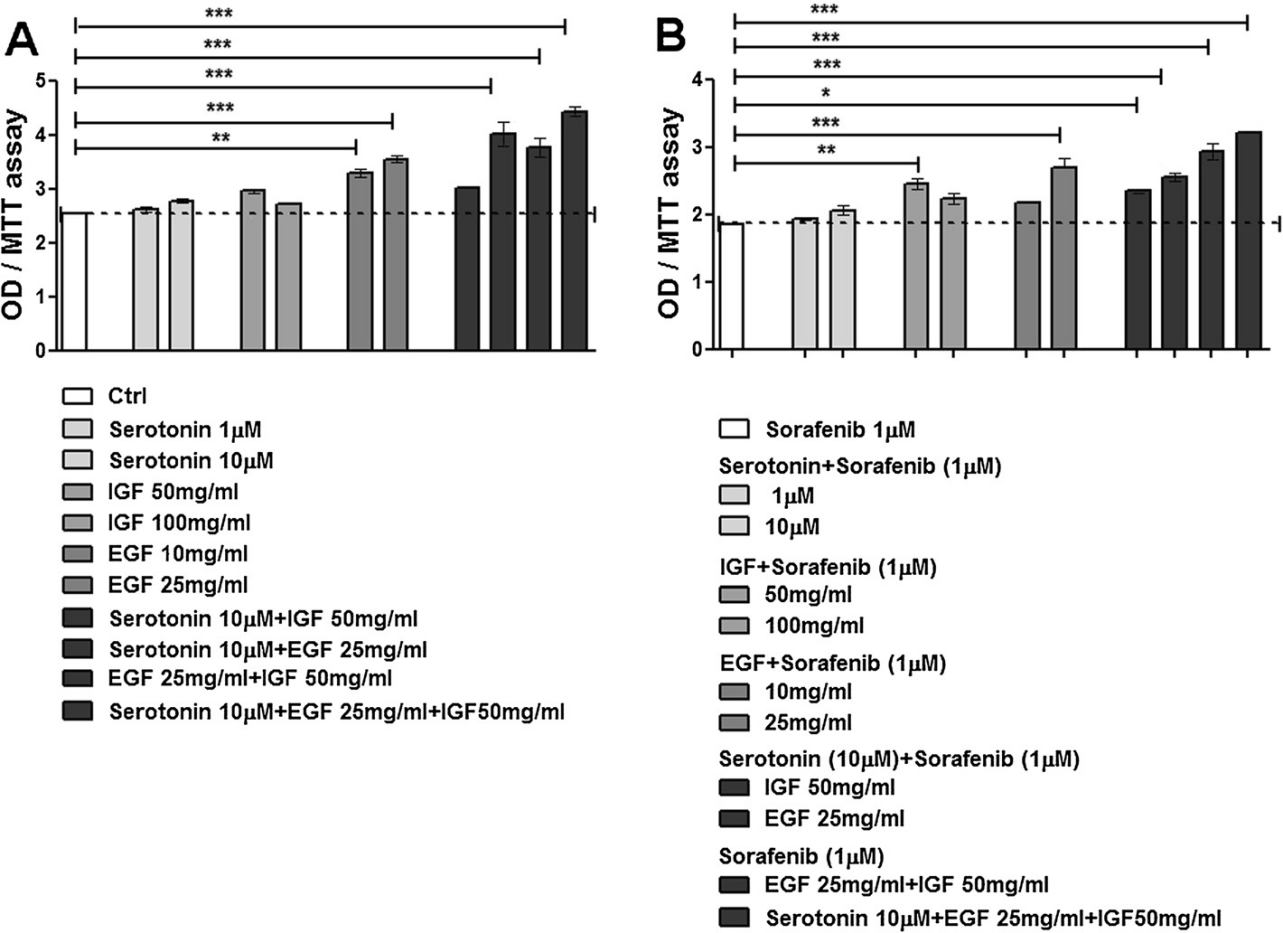
**EGF and IGF counteract the inhibitory effect of Sorafenib.** HepG2 cell line was cultured in 1% FBS medium in presence of different concentrations of serotonin, EGF and IGF-I or equivalent FBS **(A)** or incubated with the same doses of growth factors in presence of 1 μM Sorafenib **(B)**. MTT assay was assessed after 48 h. The results are expressed as mean ± SD. *p < 0.05; **p < 0.001; ***p < 0.0001.

HCC cell lines were cultured in 1% FBS in presence of different doses of serotonin (1, 10 μM), IGF (50, 100 mg/ml) and EGF (10, 25 mg/ml) alone and in combination. The effect on proliferation, evaluated by MTT assay after 48 h, was significant only with EGF, while serotonin and IGF were effective only when used in combination. Figure 5A shows the results obtained whit HepG2 cell line cultured as described above; in the graphs were plotted the effective combinations. When Sorafenib 1 μM was added to the growth factors treatments, IGF and EGF antagonized the drug inhibition of proliferation; also in this case the effect was higher when IGF and EGF were used in combination (Figure 5B).

## Discussion

We report here for the first time, the antagonizing effects of platelet extracts on growth inhibition in several HCC cell lines, that was mediated by Sorafenib or Regorafenib. Both agents were similarly antagonized by hPL. Furthermore, the previously demonstrated inhibition of AFP secretion by these drugs, was also antagonized. A main consequence of each drug is a decrease in phospho-ERK levels, secondary to Raf inhibition. hPL antagonized this early consequence of the drug action, without change in ERK levels. There was also an early and strong antagonism of the previously noted inhibitory effects of drug on phospho-p38 levels [20], and similarly for the p38 downstream target, phospho-STAT3 (Tyr and Ser). These are important molecules in mediating cell proliferation and play a role in the induction of anti-apoptosis mediators. Both Sorafenib and Regorafenib are known to increase apoptosis in treated cells. We found that this apoptosis-induction was antagonized by addition of hPL to cells that were treated with each of these two agents, as measured by both annexin V and caspase 3/7 activation.

Consistent with our findings of increased phospho-STAT3 levels, we also found an increase in the levels of anti-apoptotic Bcl-xL and survivin and a decrease in the levels of pro-apoptotic Bim and Bax, consequent to hPL action.

Due to the important role of platelets in the metastasis mechanisms of many tumors [8,21], we evaluated hPL for a possible role in stimulating cell migration or invasion. We founds that the extracts also antagonized drug-mediated inhibition of HCC cell migration and invasion on Matrigel-treated membranes. In other systems, the targeting of platelets or experimental decrease in their numbers has been shown to enhance cancer chemotherapy [22,23].

Platelets are the source of multiple growth factors, cytokines and inflammatory mediators [6].

Included among them are EGF, IGF-I, fibroblast growth factor (FGF), platelet derived growth factor (PDGF) and serotonin, the modulation of each having been shown to alter cancer chemotherapy sensitivity or resistance [24-30]. Preliminary data, obtained with several growth factors included in hPL, revealed interesting results using EGF and IGF-I. Both these factors were able to antagonized Sorafenib in a proliferation assay, in particular when used in combination. This growth induction was more evident than that observed in absence of drug, suggesting a specific interference of these growth factors with the inhibitory action of Sorafenib.

Interestingly, the clinical insulin modulator and diabetes drug, metformin [26] and the serotonin modulator Fluoxetine/Prozac that is used in depression treatment [29,30], each alter chemotherapy sensitivity in cancer cells. Multiple pathways have been found to be involved in Sorafenib-mediated growth inhibition, especially apoptosis and autophagy [19,31,32] as well as others [33-37] and several cytokines, or cytokine modulators that are produced by platelets can modulate Sorafenib activity [38]. Since Sorafenib effects have been clinically modest, several approaches are under way to enhance its actions, either on its downstream targets, or by adding inhibitors of parallel pathways in combination therapies [39]. Given the large number of candidate factors in platelets, the identification of those responsible for drug resistance is just beginning. However, FGF, IGF1 and serotonin would seem to be promising possibilities.

The recent finding that platelet inhibitors reduce hepatitis B associated experimental HCC [40] has led to new interest in the use of aspirin and other platelet inhibitors in HCC prevention, as in colon cancer prevention [41]. Thrombocytosis has been shown to be a negative prognostic factor for renal, breast, ovary, pancreas and colon cancers. Therefore, the results from this paper might be applicable to those tumor types, especially to renal cancer, since Sorafenib is also FDA-approved for treatment of renal cancer.

## Conclusion

The current results give support to the idea that platelet inhibitors might also be useful in the drug therapy of patients with unresectable HCC, provided their platelet levels and coagulation systems are normal.

## Abbreviations

HCC: Hepatocellular carcinoma; hPL: Human Platelets lysate; PVT: Portal vein thrombosis; ERK: Extracellular signal-regulated kinase; JNK: c-Jun NH2-terminal kinase; STAT: Signal transducer and activator of transcription-3; WB: Western blot; MTT: 3-(4,5-Dimethylthiazol-2-yl)-2,5-diphenyltetrazolium bromide; BrdU: 5-bromo-2′-deoxy-uridine; AFP: Alpha-fetoprotein; EGF: Epidermal growth factor; IGF-I: Insulin-like growth factor-I.

## Competing interests

The authors declare that they have no competing interests.

## Authors’ contributions

BIC involved in interpretation of clinical data, conception and design of the translational research study in vitro and wrote the first draft of the manuscript; RD, MGR and CL participated equally at the design, execution and interpretation of the experiments; NC performed Western blot experiments; GG participated in blood collection, isolation and count of platelets; CM and AC provided overall supervision for conducting the study and involved in manuscript revision and presentation. All authors read and approved the final manuscript.

## Pre-publication history

The pre-publication history for this paper can be accessed here:

http://www.biomedcentral.com/1471-2407/14/351/prepub
